# Malarial Anaemia and Anaemia Severity in Apparently Healthy Primary School Children in Urban and Rural Settings in the Mount Cameroon Area: Cross Sectional Survey

**DOI:** 10.1371/journal.pone.0123549

**Published:** 2015-04-20

**Authors:** Irene Ule Ngole Sumbele, Helen Kuokuo Kimbi, Judith Lum Ndamukong-Nyanga, Malaika Nweboh, Judith Kuoh Anchang-Kimbi, Emmaculate Lum, Yannick Nana, Kenneth K. J. Ndamukong, Leopold G. Lehman

**Affiliations:** 1 Department of Zoology and Animal Physiology, Faculty of Science, University of Buea, Buea, Cameroon; 2 Department of Biological Sciences, Higher Teachers Training College, University of Yaoundé I, Yaoundé, Cameroon; 3 Department of Biological Sciences, Faculty of Science, University of Douala, Douala, Cameroon; Institut Pasteur, FRANCE

## Abstract

**Background:**

This study examines the relative importance of living in an urban versus rural setting and malaria in contributing to the public health problem of malarial anaemia (MA) and anaemia respectively in apparently healthy primary school children.

**Methods:**

A cross-sectional study was conducted among 727 school children aged between four and 15 years living in an urban (302) and rural (425) settings in the Mount Cameroon area. Blood sample collected from each child was used for the preparation of blood films for detection of malaria parasites and assessment of malaria parasite density as well as full blood count determination using an automated haematology analyzer. Based on haemoglobin (Hb) measurements, children with malaria parasitaemia were stratified into MA (Hb<11g/dL); mild MA (Hb of 8–10.9g/dL); moderate MA (Hb of 6.1–7.9g/dL) and severe MA (Hb≤6g/dL). Evaluation of potential determinants of MA and anaemia was performed by multinomial logistic-regression analysis and odds ratios used to evaluate risk factors.

**Results:**

Out of the 727 children examined, 72 (9.9%) had MA. The prevalence of MA and anaemia were significantly higher (χ^2^ = 36.5, P <0.001; χ^2^ = 16.19, P <0.001 respectively) in children in the urban (17.9%; 26.8% respectively) than in the rural area (4.2%; 14.8% respectively). Majority of the MA cases were mild (88.9%), with moderate (5.6%) and severe MA (5.6%) occurring in the urban area only. The age group ≤6years was significantly (P <0.05) associated with both MA and anaemia. In addition, low parasite density was associated with MA while malaria parasite negative and microcytosis were associated with anaemia.

**Conclusions:**

Malarial anaemia and anaemia display heterogeneity and complexity that differ with the type of settlement. The presence of severe MA and the contributions of the age group ≤6 years, low parasite density and microcytosis to the public health problem of MA and anaemia are noteworthy.

## Background

Malaria associated anaemia represents a major public health problem in sub-Saharan Africa [1]. Its health implications, high morbidity and mortality, are more important in young children and pregnant women in malaria holoendemic and high transmission areas [1]. *Plasmodium falciparum* causes the most severe anaemia, with a significant risk of death [2]. The burden of malarial anaemia may be under estimated in malaria endemic regions in developing countries where access to appropriate health care facilities is wanting. Furthermore, only a small proportion of patients attending public health facilities receive a diagnostic test for malaria [3].

The effects of longstanding or severe anaemia can be devastating and include impairment of physical and cognitive development, especially in association with iron-deficiency; additionally, severe anaemia has been associated with an increased risk of death [4].Chronic or repeated episodes of malarial anaemia due to any *Plasmodium* species have also been associated with adverse developmental effects as well as school attendance [5,6]. Anaemia has been reported as a significant determinant of stunting [7], which is the main type of malnutrition in young children [8]. Stunting is associated with impaired cognitive development, reduced academic achievement, and decreased physical work capacity in adulthood, with negative consequences on economic development of societies [8].

The pathogenesis of malarial anaemia is multifactorial, involving the immune-and non-immune mediated haemolysis of parasitized and non-parasitized erythrocytes, bone marrow dysfunction, altered cytokine balance, nutritional deficits, and interactions with common haemoglobinopathies and erythrocyte defects such as glucose-6-phosphate dehydrogenase deficiency [9,10]. Additional variables such as endemicity of *P*. *falciparum*, antimalarial drug resistance, age, socio-demographic factors, HIV, parasitic and bacterial infections may influence the prevalence and outcomes of MA [11,12].

Severe malarial anaemia (SMA) is the most frequent severe disease manifestation in acutely febrile children with severe malaria in Cameroon [13]. SMA may be the result of an acute episode of clinical disease or a consequence of the slow and insidious process of repeated, often asymptomatic malaria infections [14] or may develop rapidly in the course of a malaria illness especially in the presence of a high parasite density [15].

Many children with malaria parasitaemia are asymptomatic particularly in malaria endemic regions. Thus, the parasitaemia frequently remains untreated for prolonged periods. The interaction between asymptomatic, low density parasitaemia and clinical episodes is complex. While higher-density is associated with symptoms (fever) and acute anaemia, asymptomatic lower-density parasitaemia may also result in anaemia, particularly if the parasitaemia persists for prolonged periods upon lack of, or ineffective treatment [16].

Despite the fact that many of Africa’s health problems are common to both urban and rural environments, the epidemiology of some diseases and the challenges to prevention and control can differ [17]. Understanding the causal factors is crucial to developing an effective intervention. This study examines the relative importance of living in an urban versus rural setting and malaria in contributing to the public health problem of MA and anaemia respectively in apparently healthy primary school children.

## Methods

### Study sites and subjects

The study was carried out in primary school children of both sexes aged 4–15 years in Limbe and rural areas in Buea situated along the slope of the Mount Cameroon area. Children were enrolled into the study only if they were; pupils in one of the chosen schools, presented a signed informed consent form from the parent/guardian and consented to the blood collection procedure.

Limbe is an urban area with high and fairly constant temperatures ranging between 25°C and 30°C on the average [18]. It also has abundant rainfall ranging from 5,500mm to 6,500mm per annum [18]. On the other hand, Buea municipality is an urban area that is closely surrounded by several rural areas. Weather records from the Cameroon Development Corporation indicate that Buea has a mean relative humidity of 80%, an average rainfall of 4,000mm and a temperature range of 18°C—27°C. There are two distinct seasons in both Limbe and Buea—a cold rainy season which spans from mid-March to October and a warm dry season with frequent light showers which runs from November to mid-March. Malaria is endemic in the Mount Cameroon area and transmission occurs all year round with an average of 1–100 malaria cases per thousand per year [19]. The first peak malaria transmission season is in April and May and the second is in October and November. These correspond with the beginning and end of the rainy season respectively. *P*. *falciparum* is the main species and *Anopheles gambiae* is the main vector species [19].

### Study design

This cross-sectional study was carried out simultaneously in the two study areas between the months of May and November, 2011 to coincide with the peak of malaria transmission season. In each school, a sensitization campaign was organized with the teachers of the schools to explain the purpose and benefits of the study before the sampling was done.

### Sampling method

The list of schools from which the sample was drawn was based on the 2011 regional summary of government, missionary and lay private schools. Schools having pupils from various backgrounds were listed in the urban and rural areas. Five (5) schools each were randomly chosen from the list of schools from both areas. Out of the 10 selected schools, head teachers from 3 Catholic schools, 3 Government and 1 private school voluntarily accepted their participation following administrative clearances from South West Regional Basic Education and Catholic Education Board. The sample size was calculated using the previous prevalence of malaria at 44.26%, anaemia at 3.83% observed in primary school children in the Mount Cameroon area by Kimbi *et al*. [20]. The sample size was determined using the formula n = Z^2^pq/d^2^ [21] where n = the sample size required, z = 1.96: confidence level test statistic at the desired level of significance, p = 44.26%: proportion of malaria prevalence, q = 1-p: proportion of malaria negative children and d = acceptable error willing to be committed. The optimum sample size was estimated as n = 379. This was adjusted by 5% considering a possible loss of samples and study areas to n = 398. To achieve the minimum sample size of 398 children each in urban and rural areas, a total of 1000 informed consent/assent forms (500 in each area) were given out to pupils. The 1000 children were randomly selected by drawing from a list of names of apparently healthy children per class. Children were considered apparently healthy if they had no physical disability, believed to be in a good state of health, had regular school attendance/ had not been absent from school due to ill health in the previous four weeks following the class records. Each school had 6 grades and the number of classes per grade varied from 1–3. Approximately 167 informed consent/assent forms were given per grade at an average of 14 per class. Only 760 pupils returned a signed informed consent/assent form.

The consent/assent form sent to parents/guardians via the children stated the purpose of the study as well as the advantages and the amount of blood that had to be collected from each child. Out of the 760 children who brought back signed informed consent forms, 727 (302 children in urban and 425 in rural areas) consented to the blood collection procedure. The children were categorized into the following age groups; ≤6, 7–10 and 11–15 years to have a reasonable representation in each age category. The investigative methods included: the use of a simple structured questionnaire to assess their socio-economic status, measurement of body temperature and laboratory analyses.

### Questionnaire

A simple structured questionnaire was administered to pupils to obtain data on key socio-economic variables including the parent/guardian’s assets. In children less than 6 years, the questionnaire was administered with the aid of the class teacher using familiar teaching aids. Based on these, all the children were classified into 3 different socio-economic status (SES) as poor (those living in plank houses, have no TV/radio, no car, use firewood kitchen and pit toilets), middle class (those living in block houses, have flush toilets, TV/radio, firewood/gas kitchen but no car) and rich (those having all what the middle class children had and a car) [22]. To avoid discrepancies, all the items were given numerical values and ranked. In cases where there was possession of items in both category, the pupils were classified based on the sum of ranks on the item possessed.

### Measurement of body temperature

Before the measure of axillary body temperature, the name, sex and age of the child was obtained from the school register. The axillary temperature of each child was measured using a clinical thermometer. A pupil was considered febrile when he/she had a body temperature ≥ 37.5°C.

### Laboratory procedure

Approximately 2mL of blood was collected from each child by venipuncture into a 2 mL sterile disposable syringe (Cathy Yougo) and dispensed into ethylenediaminetetraacetate (EDTA) tubes. Drops of whole blood were dispensed immediately on slides for the preparation of thick and thin blood films for detection and speciation of malaria parasites as described by Cheesbrough [23]. The blood samples in EDTA tubes were transported on ice blocks to the Emerging Infectious Diseases Laboratory, University of Buea for storage at 4°C. The blood samples were used for the assessment of malaria parasite density as well as full blood count.

Parasite density per μL of blood was determined on the basis of number of parasites counted per 200 leukocytes on thick blood film with reference to participants’ white blood cell counts (WBC) obtained from the complete blood count analysis. Slides were considered positive when asexual forms and/or gametocytes of any *Plasmodium* species were observed on the blood film [23]. Slides were read by two independent parasitologists and in the case of any disparity they were read again by a third parasitologist who was blinded to the findings.

The tubes containing the anticoagulated blood were rocked gently on a multitube rotator. The complete blood count: red blood cell (RBC), WBC, haemoglobin (Hb), haematocrit (Hct), platelet, mean corpuscular volume (MCV), mean corpuscular haemoglobin (MCH), and mean corpuscular haemoglobin concentration (MCHC) was run following the manufacturer’s instructions using a Beckman coulter counter (URIT 3000).

### Case definitions for the study

Fever was defined as temperature ≥37.5°C. Haemoglobin level of <11g/dL was classified as anaemia following the definitions for tropical countries by Cheesbrough [23]. Further classification was done with severe, moderate and mild anaemia having values ≤6g/dL, 6.1–8g/dL and 8.1–10.9g/dL respectively [23]. Based on the Hb measurements, children with malaria parasitaemia of any density were placed in the following categories:

- Malarial Anaemia (MA): children with Hb<11g/dL;
- Mild malarial anaemia (M*l*MA): children with Hb of 8.0–10.9g/dL;
- Moderate malarial anaemia (M*d*MA): children with Hb of 6.1–7.9 g/dL;
- Severe malarial anaemia (SMA): children with Hb ≤6.0 g/dL [12];
- Non-SMA (NSMA): children with Hb of 6.1–10.9 g/dL;Uncomplicated malaria (UM): children with Hb ≥11.0 g/dL [24].

Asymptomatic malaria (AM) was defined as the presence of *Plasmodium* with an axillary temperature of <37.5°C. Non-Malarial Anaemia (NMA) was defined as children with Hb<11g/dL and *Plasmodium* negative slide. Parasitaemia was classified as low (1–999 parasite/μL of blood), moderate (1,000–4,999 parasites/μl of blood) and high (≥5,000 parasites/μL of blood) [25]. Attributable risk (AR) defined as the amount of risk that is due to a factor was obtained as the difference in risk between exposed and unexposed children. Continuous variables were categorised as follows age groups: ≤6, 7–10 and >10 years; clinical status: clinical and asymptomatic malaria; malaria parasite status: positive and negative; parasite density: low, moderate and high; MCV: <73 fl and ≥73fl; MCHC: <320g/dL and ≥320g/dL; SES: poor, middleclass, rich and setting: urban and rural.

### Statistical analysis

Data was doubly entered into spread sheets using Microsoft Excel and validated using SPSS version 17. Analysis was done with the IBM statistical package for social sciences (SPSS) version 19 (SPSS, Inc., Chicago, IL, USA). Data was summarized into means and standard deviations (SD), and percentages were used in the evaluation of the descriptive statistics. Proportions were compared using the Chi-square test (χ^2^). Parasite densities were normalized by log_10_ transformation and geometric mean parasite densities (GMPDs) were calculated. The means of greater than two independent samples were compared using analysis of variance (ANOVA). On the other hand, means of two independent samples were compared using the t-test and Mann Whitney U test for data conforming to normal and those not conforming to normal distribution respectively. Attributable Risk of anaemia caused by malaria (AR %) was calculated accordingly: [*(n*_*1*_ *m*_*0—*_ *n*_*o*_ *m*_*1*_ *) / n (n*_*0*_ *+m*_*0*_ *)*] x 100 where n_0_ = anaemic children without malaria, n_1_ = anaemic children with malaria, whereby n_0_ + n_1_ = n; m_0_ = non-anaemic children without malaria, and m_1_ = non-anaemic children with malaria, whereby m_0_ + m_1_ = m [26]. Evaluation of potential determinants of MA and anaemia was performed by multinomial logistic-regression analysis. Stepwise backward selection was first performed to eliminate potential confounders and the final models included those factors that retained statistical significance. In the anaemia model, all the 727 children examined were included in the analysis, while in the MA model, only the 245 malaria positive cases were entered into the model. A forward stepwise method of entry was used in the final two models where variables such as sex and altitude were forced into the models. The order of forward entry of categorical variable was sex, altitude, age groups, malaria parasite status, parasite density category and MCV. The odds ratios (OR) and 95% confidence intervals (CI) computed was used to evaluate risk factors associated with MA and anaemia. Significant levels were measured at 95% CI with significant differences recorded at P < 0.05.

### Ethical considerations

Before commencement of the study, an ethical clearance was obtained from the South West Regional Delegation of Public Health while administrative clearances were obtained from the Regional Delegation of Basic Education as well as from the Catholic Education Board. Children participated in the study if a parent or guardian signed the informed consent form. The parents or guardian and their children were informed that their participation in the study was voluntary and they could withdraw at any time without any explanation.

## Results

### Socio-demographic and clinical characteristics of the study population

Out of the 727 primary school children evaluated for malaria related anaemia in the urban and rural settings in the Mount Cameroon area, 443(60.9%, CI = 57.3–64.4%) were in the 7–10 years age group and majority (71.5%, CI = 68.1–74.7%) of them were of poor SES. The proportions of males (47.8%, CI = 44.3–51.5%) and females (52.2%, CI = 48.6–55.9%) in the study population were comparable as seen in Table 1. Overall, the prevalence of malaria parasitaemia, AM, and UM was33.8% (CI = 30.4–37.2%), 21.9% (CI = 19.0–25.0%) and 23.8% (CI = 20.8–27%) respectively. Anaemia was prevalent in 19.8% (CI = 17.1–22.9%) of the children examined, while 9.9% (CI = 7.9–12.3%), 9.9% (CI = = 7.9–12.3%) and 15.2% (CI = 12.7–17.9%) had MA, NMA and microcytosis respectively. The prevalence of microcytosis was significantly higher (χ^2^ = 37.1, P <0.001) in children who were malaria parasite positive (26.5%, CI = 21.4–32.4%) when compared with their negative counterparts (9.4%, CI = 7.1–12.3%).The AR of anaemia caused by malaria was 24.5%.

**Table 1:** Demographic, clinical and erythrocytic characteristics in the different settings.

Characteristic		Settings			Test
	Category	Urban	Rural	All	P—Value
Number of children		302	425	727	
Sex	Female (%)	156 (51.7)	224 (52.7)	380 (52.2)	0.11‡
	Male (%)	146 (48.3)	201 (47.3)	348 (47.8)	0.75
Age group (years)	≤ 6 (%)	83 (27.4)	62 (14.6)	145 (19.9)	30.48‡
	7–10 (%)	185 (61.1)	258 (60.7)	443 (60.9)	<0.001
	>10 (%)	25 (11.6)	105 (24.7)	140 (19.2)	
Mean age (years)		7.96 ± 2.13	9.09 ± 2.18	8.62 ± 2.23	6.95§
					<0.001
SES, n (%)	Poor	205 (68)	314 (73.9)	519 (71.5)	5.62‡
	Middle class	63 (20.8)	60 (14.1)	123 (16.9)	0.06
	Rich	34 (11.2)	51 (12)	85 (11.7)	
Prevalence (%) of Fever (n)		33.7	27.3	29.9	3.42‡
		(102)	(116)	(218)	0.06
Prevalence (%) of malaria parasitaemia (n)		60.3	14.8	33.8	163.2‡
		(182)	(63)	(245)	<0.001
Prevalence (%) of AM (n)		38.4	10.1	21.9	81.9‡
		(116)	(43)	(159)	<0.001
Prevalence (%) of UM (n)		42.5	10.6	23.8	99‡
		(128)	(45)	(173)	<0.001
GMPD/ μL of blood (Range)		233	442	275	2.552§
		(26–92,880)	(18–220,000)	(18–220,000)	0.013
Mean Hb (SD) in g/dL (Range)		11.50 (1.56)	12.08 (1.17)	11.84 (1.37)	5.67§
		(5–16.3)	(5.1–15.8)	(5–16.3)	<0.001
Prevalence (%) of Anaemia (n)		26.8	14.8	19.8	16.19‡
		(81)	(63)	(144)	<0.001
Prevalence (%) of MA (n)		17.9	4.2	9.9	36.5‡
		(54)	(18)	(72)	<0.001
Prevalence (%) of microcytosis (n)		34.1	1.6	15.2	145.42‡
		(103)	(7)	(110)	0.001
Prevalence (%) of NMA (n)		9.0	10.6	9.9	0.52‡
		(27)	(45)	(72)	0.47

‡ χ^2^

§ Statistical significance determined by t-test.

### Influence of setting

Children in the urban set up had a significantly higher prevalence of malaria parasitaemia, AM, UM, anaemia, MA and microcytosis when compared with those in the rural area as shown in Table 1. The geometric mean parasite density (GMPD)/ μL of blood and mean Hb (g/dL) concentration were significantly higher (P = 0.013; P <0.001) in children in the rural (442 parasites/μL of blood; 12.08g/dL respectively) than those in the urban (233 parasites/μL of blood; 11.50g/dL respectively) area (Table 1).

Parasitaemia of low density was significantly higher in children in the urban area (91.2%, CI = 86.2–94.5%) than the rural (71.4%, CI = 59.3–81.1%), while the prevalence of moderate and high parasite density was higher in children in the rural (17.5%, CI = 10–28.6%; 11.1%, CI = 5.5–21.2% respectively) than the urban area (7.1%, CI = 4.2–11.8%; 1.7%, CI = 0.6–4.7% respectively) as shown in Table 2.

**Table 2:** Prevalence (%) of the different categories of parasite density, MA and anaemia in the two settings.

Parameter		Urban	Rural	Total	χ^2^
	Category	% (n)	% (n)	% (n)	P-value
Parasite density	Low	91.2 (166)	71.4 (45)	86.1 (211)	17.5
	Moderate	7.1 (13)	17.5 (11)	9.8 (24)	<0.001
	High	1.7 (3)	11.1 (7)	4.1 (10)	
MA	M*l*MA	85.2 (46)	100 (18)	88.9 (64)	3.0
	M*d*MA	7.4 (4)	0.0	5.6 (4)	0.22
	SMA	7.4 (4)	0.0	5.6 (4)	
NSMA		16.6 (50)	4.2 (18)	9.4 (68)	31.4
					<0.001
Anaemia	Mild	88.9 (72)	98.4 (62)	93.1 (134)	5.2
	Moderate	4.9 (4)	0.0	2.8 (4)	0.07
	Severe	6.2 (5)	1.6 (1)	4.2 (6)	

The prevalence values of MA and anaemia were significantly higher (χ^2^ = 36.5^,^ P <0.001; χ^2^ = 16.19^,^ P <0.001 respectively) in children in the urban (17.9%, CI = 14–22.6%; 26.8%, CI = 22.2–32.2% respectively) than in the rural area (4.2%, CI = 2.7–6.6%; 14.8%, 11.8–18.5% respectively). In the urban, but not in the rural area, the prevalence of MA decreased with improved SES (Poor = 18.4%, CI = 13.7–24.3%; middle class = 17.5%, CI = 10.04–28.6% and rich = 14.7%, CI = 6.5–30.1%). In children in the urban area, the AR of anaemia caused by malaria was 15.7% while in children in the rural area it was 16.1%.

Overall, the prevalence of SMA in children in the urban and rural areas was 1.3% (CI = 0.–3.4%) and 0% while that of severe anaemia was 1.7% (CI = 0.7–3.8%) and 0.2% (CI = 0.04–1.3%) respectively. Although all the MA cases in the rural area were mild, the prevalence of mild to SMA did not differ significantly (P >0.05) between children in the urban and the rural areas. However, the prevalence of NSMA was significantly higher (P <0.01) in children in the urban than in the rural area (Table 2).

Malarial anaemic children in the rural area had significantly higher mean Hct (33.9%) and MCV (85.8 fl) than those in the urban areas (27.7% and 73.8 fl respectively). On the other hand children in the urban area had significantly higher MCHC (338 g/dL) than those in the rural area (302.7 g/dL) as shown in Table 3.

**Table 3:** Comparison of mean (SD) red cell indices in malarial anaemic children in the different settings.

Parameter		Urban	Rural	Total	P-value‡
N		54	18	72	-
Hb in g/dL	Mean (SD)	9.6 (1.7)	10.2 (0.6)	9.7 (1.6)	0.4
	CI	9.1–10.1	9.9–10.5	9.32–10.1	
RBC × 10^12^/L	Mean (SD)	3.8 (0.9)	3.97 (0.5)	3.8 (0.8)	0.5
	CI	3.6–4.0	3.7–4.2	3.6–4.0	
Hct in %	Mean (SD)	27.7 (5.9)	33.9 (3.7)	29.3 (6)	< 0.001
	CI	26.1–29.3	32.1–35.7	27.9–30.7	
MCV in fl	Mean (SD)	73.8 (72)	85.8 (6)	76.8 (8.6)	< 0.001
	CI	71.8–75.8	82.8–88.8	74.8–78.8	
MCHC in g/dL	Mean (SD)	338 (22.4)	302.7 (21.3)	329.2 (26.8)	< 0.001
	CI	331.9–344.1	292.1–313.3	322.9–335.5	
MCH in pg	Mean (SD)	26.4 (10.9)	25.9 (2.5)	26.3 (9.5)	0.1
	CI	23.4–29.4	24.7–27.1	24.1–28.5	

‡ Statistical significance determined by Mann-Whitney U test.

### Age dependent pattern

Children in the ≤6 years age group had the highest prevalence of malaria parasites (42.8%, CI = 35–50.9) and GMPD (407, 29–12,760/ μL of blood) when compared with the other age groups. The differences were statistically significant (P = 0.003, P = 0.03 respectively) as seen in Table 4. The prevalence of anaemia and MA was significantly highest (P <0.001) in children of the youngest (≤6) age group (34.5%, CI = 27.2–42.5%; 20.1%, CI = 14.3–27.3% respectively).

**Table 4:** Malaria, anaemia and MA as affected by age.

Parameter		Age group (years)			Total	Test
		≤6	7–10	>10		P value
N		145	443	139	727	-
Prevalence of Malaria	%	42.8	34.1	23.0	33.7	12.4‡
	(n)	(62)	(151)	(32)	245	0.002
GMPD/μL of blood	GMPD	407	251.4	195.3	247.8	3.6^§^ ʄ
	(n)	(62)	(151)	(32)	(245)	0.03
	Range	29–12,760	18–220,000	27–9,286	18–220,000	
Prevalence of anaemia	%	34.5	17.9	10.8	19.8	27.8‡
	(n)	(50)	(79)	(15)	(144)	<0.001
Hb in g/dL	Mean (SD)	11.2 (1.8)	11.9 (1.2)	12.4 (1.2)	11.8 (1.4)	33.9ʄ
	95% CI	10.9–11.5	11.8–12	12.2–12.6	11.7–11.9	<0.001
Prevalence of MA	%	20.1	8.6	3.6	9.9	24.1‡
	(n)	(29)	(38)	(5)	(72)	<0.001

‡ χ^2^

ʄ ANOVA

^§^ Log transformed parasitaemia was used in analysis.

### Malaria parasite density

**Fig 1:**
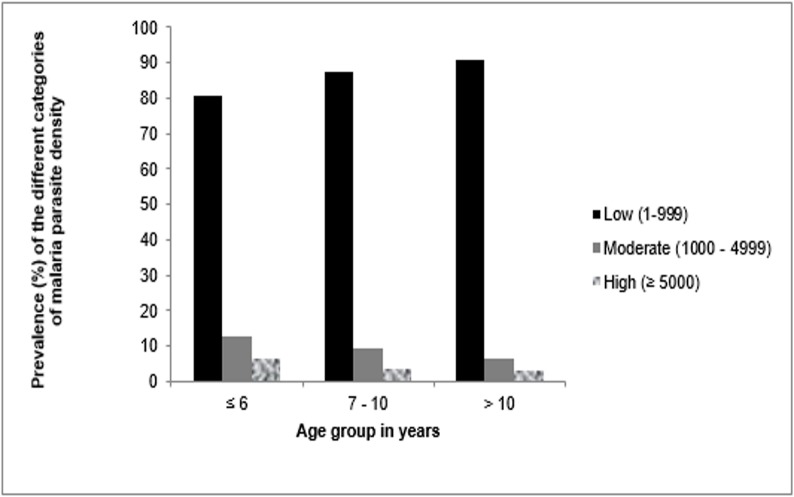
Variation in prevalence of the different categories of parasite densities in the different age groups.

The prevalence of low, moderate and high parasitaemia in the population was 86.1% (CI = 81.2–89.9%), 9.8% (CI = 6.7–14.2%) and 4.1% (CI = 2.2–7.4%) respectively. The variations in prevalence of the different categories of parasite densities were comparable (P = 0.64) in the different age groups as shown in Fig 1.

**Fig 2:**
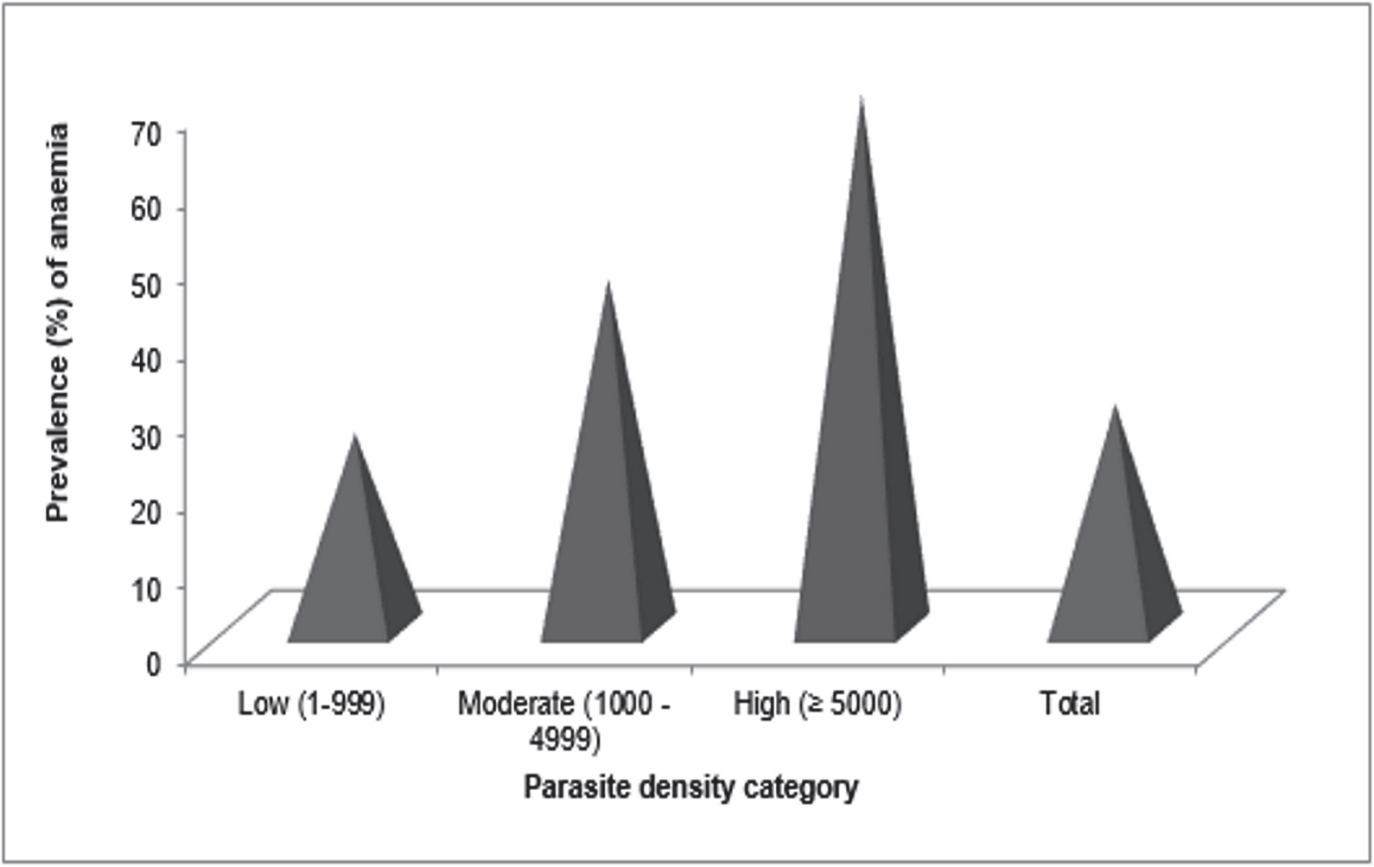
Prevalence (%) of anaemia as affected by parasite density.

Out of the 245 children who were malaria parasite positive, 72 (29.3%, CI = 24–35.4%) had MA. Anaemia prevalence increased with the parasite density category. The prevalence of anaemia was significantly highest (χ^2^ = 12.5, P = 0.002) in children with high parasite density (Fig 2).

### Haematological values

Children with MA had a significant reduction (P <0.01) in mean Hct and RBC counts when compared with their NMA counterpart. On the other hand, the MCHC was significantly higher (P <0.001) in children with MA than those with NMA as shown in Table 5. Although not statistically significant, the mean Hb concentration, MCV, WBC and platelet counts were lower in children with MA than their NMA counterparts (Table 5).

**Table 5:** Mean haematological indices in malarial anaemic and non malarial anaemic children.

Variable				t—test	Mean difference
	Status	N	Mean (SD)	P—value	95% CI
Hb (g/dL)	MA	72	9.7 (1.6)	-1.86	-0.42
	NMA	72	10.2 (1.1)	0.07	-0.87–0.03
Hct (%)	MA	72	29.3 (6)	-3.8	-3.43
	NMA	72	32.7 (4.8)	<0.001	-5.23–-1.65
RBC × 10^12^/L	MA	72	3.8 (0.8)	-2.71	-0.32
	NMA	72	4.2 (0.6)	0.008	-0.55–-0.09
WBC × 10^9^/L	MA	72	5.7 (2.9)	-0.47	-0.19
	NMA	72	5.9 (2)	0.64	-1—0.62
MCV (fl)	MA	72	76.8 (8.6)	- 1.57	- 2.39
	NMA	72	79.2 (9.6)	0.12	-5.39–0.61
MCH (pg)	MA	72	26.3 (9.5)	1.52	1.75
	NMA	72	24.6 (2.4)	0.13	-0.53–4.04
MCHC (g/dL)	MA	72	329.2 (26.8)	3.65	16.58
	NMA	72	312.6 (27.8)	<0.001	7.59–25.58
Platelet × 10^9^/L	MA	54	305.4 (162.9)	612.5	-
	NMA	27	347.6 (180.5)	0.24‡	

‡ Statistical significance determined by Mann-Whitney U test.

### Factors associated with MA and anaemia

The different categories of age groups, sex, clinical status, parasite density, malaria parasite status, MCV, MCHC, SES and setting were entered into the stepwise multinomial logistic regression model. The age group ≤6years was significantly (P <0.05) associated with both MA and anaemia. In addition, parasitaemia density < 1,000 parasites/μL of blood (low) was associated with MA while malaria parasite negative and microcytosis were associated with anaemia (Table 6).

**Table 6:** Multinomial analysis of risk factors associated with MA and anaemia.

Parameter		MA			Anaemia		
		β-value	P- value	OR (95% CI)	β -value	P- value	OR (95% CI)
Sex	(Female)	0.01	0.98	1.01 (0.56–1.8)	0.05	0.81	1.05 (0.72–1.53)
Age groups (years)	≤ 6	-1.36	0.02	0.26 (0.08–0.78)	-1.23	< 0.001	0.29 (0.15–0.56)
	7–10	-0.51	0.33	0.06 (0.21–1.70)	-0.46	0.14	0.63 (0.35–1.15)
Altitude		0.33	0.89	1.03 (0.63–1.69)	-0.03	0.85	0.97 (0.74–1.28)
Malaria parasite density category	Low (<1,000)	1.92	0.01	6.84 (1.56–29.9)	ND	ND	ND
	Moderate (1,000–4,999)	1.06	0.2	2.9 (0.57–14.8)	ND	ND	ND
Malaria parasite	(Negative)	ND	ND	ND	0.66	0.004	1.93 (1.24–3.03)
Microcytosis	(MCV <73 fl)	ND	ND	ND	-0.8	0.002	0.45 (0.27–0.75)

ND, not determined as it is redundant in the multinomial model.

## Discussion

Data on malarial anaemia is lacking in most transmission settings even though anaemia has been reported [27] as a common haematological state among malaria subjects in the Mount Cameroon area. The lack of up to date epidemiological data in many parts of Cameroon is a serious handicap towards attempting to evaluate the impact of control measures against malaria morbidity. The children evaluated in this study consisted of apparently healthy pupils with regular school attendance as opposed to the hospital—based studies in other malaria endemic regions [12,24].

Urbanization may alter the frequency and transmission dynamics of malaria. The higher prevalence of malaria in urban (60.6%) when compared with the rural (14.8%) area may be attributed to favourable climatic conditions such as high temperatures and rainfall which promote the rapid growth of the anopheline vectors. Malaria transmission in urban areas varies according to a location (e.g., altitude, proximity to a sea, river, or floodplain), climate, land use, human movement patterns, socioeconomic factors, local vector species, vector breeding sites, waste management, and local malaria intervention programs [28]. The presence of ‘pockets’ of bushy areas in different parts of the town around houses, coupled with urban agriculture may provide optimal conditions for vector breeding, leading to a higher risk of malaria transmission in this vicinity.

Observations from the study revealed that children from the rural area had a significantly higher level of haemoglobin than their urban counterparts even though the difference was not statistically significant when only malarial anaemic children in the different settings were compared. The higher mean Hb concentration in children in the rural area coupled with a higher GMPD/μL of blood probably reflects the impact of lesser malaria transmission intensity when compared with the urban area. In areas of endemicity, the rate of exposure to infected mosquitoes has been correlated with the density but not the prevalence of parasitemia [29]. In addition parasite density is linked to disease, and diminished parasite counts almost certainly contribute to diminished risk of disease [30].The observed higher prevalence of MA in children in the urban area may be attributed to the significantly higher prevalence of malaria parasitaemia in the area and the impact of the low parasite density on haemoglobin levels. Majority of children in the urban area had low parasite density. As indicated by the model, children with low parasite density were 7times at odds (OR = 6.84) of developing MA than their other counterparts. However, the overall effect of the location of the children in the different setting on the risk of malarial anaemia was non-significant.

The overall prevalence of anaemia (19.8%) was higher than that obtained by Kimbi *et al*. [20] in school children in the Mount Cameroon area (3.8%) in 2011, but lower than that of Gudo *et al*. [31] in Mozambique (>50%) in 2005. In addition, the prevalences of M*d*MA, SMA, NSMA, moderate and severe anaemia were higher in children in the urban than rural area even though, the AR of anaemia caused by malaria were comparable in both areas. The presence of SMA in school children in the urban area with regular school attendance is a cause for concern as SMA increases the risk of death in children [32]. Severe malarial anaemia is the primary clinical manifestation of severe childhood malaria in areas of intense and sustained transmission [11,33] and both symptomatic and asymptomatic malaria contribute substantially to anaemia in endemic regions [34,35]. The overall prevalence of 21.9% of asymptomatic malaria in the study population may have contributed substantially to the presence of SMA. The observed prevalence of SMA may be the consequence of a slow and insidious process of repeated often asymptomatic malaria infection [14] or impaired and/or ineffective erythropoiesis [33].

The prevalence of MA decreased with improved socio-economic status in the urban area, but not in the rural highlighting the problem of unregulated rapid urban growth on the health of the population. Buea municipality has experienced a rapid growth in infrastructure, population and socio-economic changes within the last decade although the surrounding area from which the study population was drawn still remains largely rural. This has also been accompanied by the institution of a monthly clean-up campaign to improve on the environmental conditions. In line with Tatem *et al*. [36] and Kimbi *et al*. [37], the enhanced malaria control measures as well as the process of urbanization may have resulted in profound socio-economic and landscape changes that reduced malaria transmission. A reduction in malaria transmission is likely to lead to better haematological values.

The decreased prevalence of malaria parasite, GMPD/μL of blood, anaemia and MA with an increase in the age group is not unusual as age has been associated with a decrease in disease severity [30]. The age group ≤ 6 years was the only significant factor associated with the risk of having anaemia or MA as indicated in the model in Table 6. The age related decrease in malaria related disease manifestations is consistent with the observation that the speed of acquired anti-disease immunity depends on the frequency of parasite exposure from birth [30].

Parasite density may be used as a surrogate marker of transmission intensity in malaria endemic areas [38]. The significant difference in the prevalence of the various parasite density categories in the urban and rural areas probably reflects the differences in the transmission intensity in both areas. Results from the study revealed that majority of children (91.2%) with malaria parasitaemia living in the urban area had less than 1000 parasites/μL of blood (low), while those living in the rural area recorded a higher prevalence of moderate (17.5%) and high (11.1%) parasite density than those in the urban area (7.1% and 1.7% respectively). As previously reported by Kitua *et al*. [39], the maintenance of low parasite density in both areas may have been fundamental to the survival of the children and may have contributed in reducing the development of SMA and hence morbidity. Nonetheless, SMA is the primary clinical manifestation of severe childhood malaria in areas of intense and sustained transmission [33]. Also, the presence of moderate to SMA in children with low parasitaemia living in the urban area is not unusual as parasitemia among patients with severe malarial anaemia have a propensity to be low compared to that among less anaemic patients [30]. Furthermore, findings from the study lend more support to this as the low parasite density category was shown by the regression model to be a significant risk factor for malarial anaemia.

Although the prevalence of anaemia increased significantly with the parasite density category (Fig 2), the logistic regression model did not associate the high parasite density category with malarial anaemia (Table 6). However, a cause and effect relationship between *P*. *falciparum* and anaemia has been highlighted by Biemba *et al*. [40] with severity of anaemia increasing with parasite counts in hospitalized children. Findings from this study in apparently healthy school children did not demonstrate such a significant relationship. None of the children with parasite counts >5000 parasites/μL of blood had severe or moderate anaemia.

Even though observation from the study suggests parasite density of the lowest category to be significantly associated with malarial anaemia, other causes of anaemia cannot be excluded. However, it is possible that the lower- parasitaemia density could have kept the children in a sub-optimal haematologic state that prevented them from attaining the more favourable Hb concentrations of parasite-free children. McElroy *et al*. [41] reported children with lower Hb due to persistent lower-density parasitaemia to be at increased risk for severe anaemia due to their reduced capacity to buffer additional parasitologic insult. It is unclear how long these children were parasitaemic but findings from the study attest to this as the GMPD/μL of blood decreased with an increase in the severity of anaemia.

The significant reduction in some red cell indices (RBC and Hct) and increase in MCHC observed in children with MA is not unexpected. Haematological abnormalities are considered a hallmark in infection with *P*. *falciparum*. Thrombocytopenia and anaemia have been commonly observed in malaria infected individuals in other studies [27,42,43]. Findings from the study further revealed significantly lower haematological values (Hct, MCV) in children in the urban areas when compared with their rural counterparts. This observation may be a reflection of the influence of malaria parasites on haematological indices following the subtle impact of urbanisation, climatologic and geographic conditions that are favourable to the mosquito vector and hence malaria parasite transmission. Furthermore, while MA was significantly higher in children from the urban area (Table 1), the prevalence of NMA was comparable in both areas.

Findings from the study are consistent with previous reports that have associated microcytosis with lower haemoglobin levels [44, 45]. Microcytosis has also been reported [46] as a common morphological finding in children with anaemia in the Mount Cameroon area. Although microcytosis was significantly higher in children who were malaria parasite positive and those from the urban area, the multinomial regression model revealed microcytosis to be a significant risk factor related to anaemia rather than MA. This is in line with Cornet *et al*. [44] who also reported microcytosis to be a significant factor related to anaemia. The presence of microcytosis even in those who were malaria parasite negative probably points out the role of undiagnosed iron deficiency in some of the children and thalassaemia which is common in sub-Saharan Africa [47]. However findings by Fowkes *et al*. [48] assert that a lower concentration of Hb per erythrocyte and a larger population of erythrocytes may be a biologically advantageous strategy against the significant reduction in erythrocyte count that occurs during acute infection with *Plasmodium falciparum* and may reduce the risk of anaemia by other *Plasmodium* species, as well as other causes of anaemia. Also, they further stated other host polymorphisms that induce an increased erythrocyte count and microcytosis may confer a similar advantage.

The finding in the overall population that children who were malaria parasite negative were significantly associated with anaemia is unexpected and in contrasts with the main literature [1,34,35]. Given the AR of anaemia caused by malaria observed in this study (24.5%), which is higher than the over 6% observed in Accra and Kumasi, Ghana [49], the generalization of being malaria parasite negative as a risk factor of anaemia is limited. Even though a substantial proportion of anaemia in the population could be due to other factors that were not investigated, there is also likelihood that these apparently healthy children could be harbouring low-level sub-microscopic parasitemias which is important in the onset and maintenance of immunity/premonition [30] whose impact cannot be ignored.

## Conclusions

Malarial anaemia and anaemia display heterogeneity and complexity that differ with the urban setting hence, its implication in control efforts. Malarial anaemia decreased with improved socioeconomic status in children living in urban areas accentuating the need for a regulated growth in urban areas. The presence of SMA and the contributions of age group ≤ 6 years, low parasite density and microcytosis to the public health problem of MA and anaemia in healthy children with regular school attendance are noteworthy. Nonetheless, further investigation on the contribution of microcytosis, iron deficiency and thalassaemia to these health problems are crucial.
